# Cherenkov emission in realistic optical body phantoms to study effects of skin tone on imaging delivery technique

**DOI:** 10.1088/1361-6560/ae237d

**Published:** 2025-12-05

**Authors:** Jeremy E Hallett, Siddharth Kulkarni, Aubrey Parks, Jennie Crosby, Carri Glide-Hurst, Jacob Wudtke, William Ware, Brian W Pogue

**Affiliations:** 1 Department of Medical Physics, University of Wisconsin-Madison, Madison, WI, United States of America; 2 Department of Human Oncology, University of Wisconsin-Madison, Madison, WI, United States of America; 3 UW Health, Madison, WI, United States of America; 4 DoseOptics LLC, Lebanon, NH, United States of America

**Keywords:** Cherenkov, surface guided imaging, radiation therapy, phantoms

## Abstract

*Objective.* In radiotherapy, Cherenkov light emitted from tissue originates predominantly from depths of 0–5 mm within the body, and subsequently darker skin can reduce the observed signal passing through the epithelium. The goal of this study was to assess the spatial correlation between patient near-surface dose and Cherenkov emission with varying patient skin tones and radiotherapy delivery techniques. The work focused on tissue phantoms that recapitulated the layered structure of human melanin pigment in skin allowing for a systematic study difficult to perform in humans. *Approach.* Six tissue-like, layered silicone phantoms following the progressive Fitzpatrick skin scale were created using a thermoformed mold of a torso phantom. Pigments were applied in thin silicone coatings to simulate the epidermis. Each skin was successively placed on the phantom and computed tomography-scanned for whole breast irradiation treatment planning. Plans were delivered to each phantom for: two tangent fields, dynamic field-in-field, and volumetric modulated arc therapy (VMAT), with online Cherenkov imaging. *Main Results.* The measured Cherenkov intensity showed a linear trend with luminosity (*r*^2^ $ \approx $ 0.97) and a melanin index (MI) range consistent with literature (MI range 37–120). Cherenkov image shape comparison with the expected surface dose maps showed a dice score range of 0.93–0.95 for all treatment plans on lighter skin phantoms. For the darkest skin type (MI = 120), the dice score decreased to ∼0.88 for the tangent and field-in-field plans and to ∼0.80 for VMAT. When disagreement was found it tended to be in the lowest dose edges of the field. *Significance.* Large MI values (type VI) result in lower Cherenkov-to-plan agreement as compared to measurements made with lighter skin tone phantoms, especially when imaging VMAT plans which show reduced optical flux. These results highlight the need for strategies that increase captured optical signal for situations where the Cherenkov light is attenuated by high melanin.

## 1. Introduction

It has been well established in literature that the skin tone of a patient can lead to limitations in the performance of optical measurements in Medicine. Kapoor *et al*, reported that surface monitoring systems used for verifying patient position during external beam radiation therapy demonstrated reduced patient positioning accuracy and reliability when used to measure dark pelvis phantoms of high Fitzpatrick skin type (2024). AAPM task group 302 recommends that clinics perform surface monitoring QA with light and dark phantoms to assess this effect (Al-Hallaq *et al* 2022) and Covington *et al* demonstrated the use of 3D printed anthropomorphic phantoms for this purpose (2025). Beyond radiotherapy, pulse oximetry, an optical method used to measure oxygen saturation in a blood, is more likely to overestimate the oxygen saturation levels of patients with darker skin (Setchfield *et al* 2024).

Recently, the emission of Cherenkov radiation during external beam radiation therapy has been used to verify the location of dose deposition on a patient’s skin surface (Chen *et al* 2022, Alexander *et al* 2023). When high energy charged particles, such as electrons, travel faster than the phase velocity of light in the medium of interest, Cherenkov light is produced based upon the medium’s index of refraction and the particle’s velocity (1937). Assuming an index of refraction of 1.4, the threshold secondary electron energy for Cherenkov production in human skin tissue is 0.219 MeV (Axelsson *et al* 2011). Most photon–matter interactions at radiotherapy energies result in the production of secondary electrons beyond this threshold. This emitted Cherenkov light can be captured by an intensified complementary metal–oxide–semiconductor camera gated to the pulse structure of the medical linear accelerator and used for treatment monitoring.

It has been established that the intensity of Cherenkov photons that are transmitted out of the patient is contingent on the concentration of melanin in the outer layer of the skin (Decker *et al* 2022, Wickramasinghe *et al* 2023). Patients with darker skin tone exhibit more light attenuation which leads to lower intensity images, and in the most extreme cases it might also alter the treatment field shape measured as the escaping photon spectra is dependent on the tissue optical properties (Zhang *et al* 2017) effecting the low signal field edges. The variance in skin tone across a patient can be high, and the angle of tissue relative to the radiation beam and camera can affect the emitted light as well. Both factors can make light emission heterogeneous, and decrease the accuracy of Cherenkov *in-vivo* dosimetry.

While color luminosity imaging and computed tomography (CT) corrections have been shown to improve the correlation between Cherenkov and dose for static beam treatments (Hachadorian *et al* 2022), at present, limited work has been conducted to characterize the loss of beam spatial localization when also considering dark skin-tones. It has been suggested that additional factors can further reduce the detected intensity, such as complex treatment plans using a high degree of modulation (Parks *et al* 2025). Cherenkov imaging is signal limited. Therefore, any reduction of light emission may result in a failure to detect radiation field abnormalities.

The focus of this study was to systematically quantify the spatial accuracy of surface Cherenkov emission compared against the planned surface dose from the treatment plan. Availability of Cherenkov data from a representative cohort of patients is currently limited due to the technology introduction phase and the variation between plans of individual patients. Thus, a large part of this work was dedicated to creating tissue mimicking phantoms that would allow for a systematic study of the same treatments in phantoms of different skin tone. These phantoms can be treated many times under various conditions and treatment plans without any dose limit.

## 2. Methods

### 2.1. Phantom development & pigment measurement

**Figure 1.**
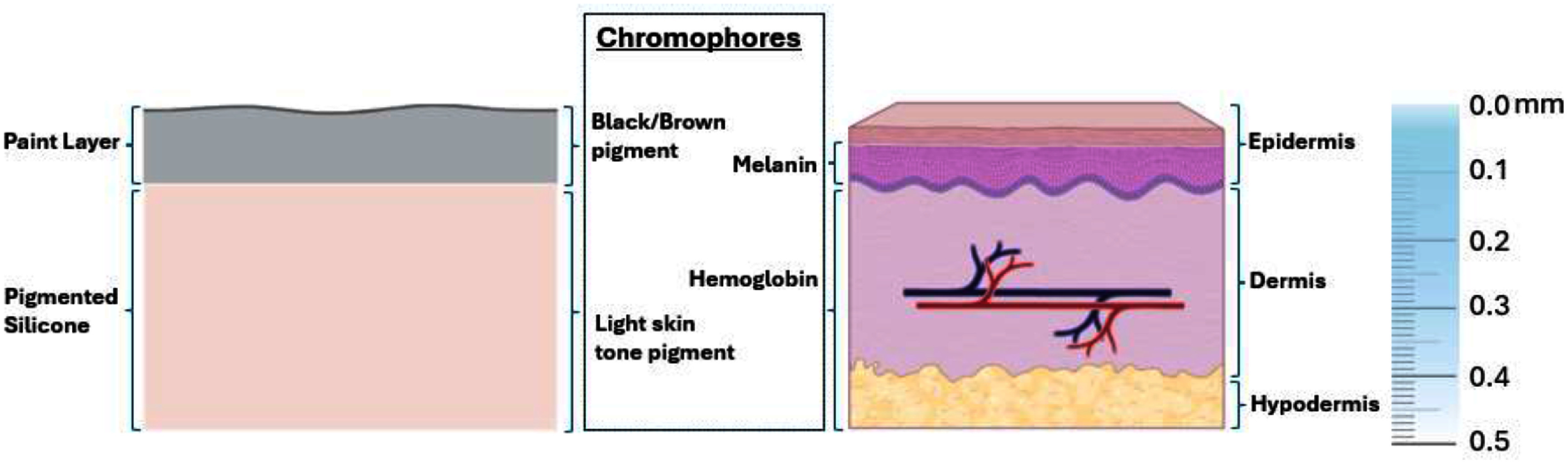
The tissue optical properties of skin were mimicked using a pigmented silicone bulk phantom material to act as the dermis and hypodermis layers of the skin with a layer of pigmented paint applied at the surface to take the place of the epidermis. Brown and black pigment replaced melanin while hemoglobin was simulated with light skin tone pigment. The depth limit of Cherenkov light escaping the skin is close to 5 mm as the light attenuates exponentially (Zhang *et al* 2013). The gradient on the depth scale illustrates how the amount of Cherenkov light escaping the tissue decreases with depth.

A set of six silicone skin mimicking phantoms were produced for this work, each simulating different levels of skin melanin concentration. An anthropomorphic bariatric cardiopulmonary resuscitation (CPR) phantom was used as the base of the phantom with the silicone skin placed on top. Silicone demonstrates similar optical properties to that of human skin tissue and is a useful tool for Cherenkov imaging studies. The density of silicone is nearly identical to that of human tissue with densities of roughly 1.07 g cm^−3^ (Smooth-On) and 1.04 g cm^−3^ (Akhlaghi *et al* 2015) for silicone and soft tissue, respectively. Additionally, silicone has an effective atomic number (${Z_{{\text{eff}}}})$ close to that of soft tissue. Moldable silicone has ${Z_{{\text{eff}}}} = 10.7$ (Aldosary *et al* 2022) while soft tissue exhibits ${Z_{{\text{eff}}}} = 7.35$. It should be noted that this difference in *Z* may result in slightly more photon interactions in the silicone phantom due to an increased probability of pair-production and photo–electric interactions, however, the effective atomic number and particle energies used in this study result in the Compton effect being the dominant interaction. The Compton scattering cross section is dependent on the number of electrons per unit mass and not the atomic number. Silicone is known to have a similar number of electrons per gram compared to soft tissue (Shrimpton 1981, Du *et al* 2019), therefore the number of secondary electrons produced that can create Cherenkov emission is similar between soft tissue and the silicone phantom Despite this Compton dominance, the slight increase in pair production with larger *Z* should be considered when interpreting the results as it suggests slightly more Cherenkov production in the higher *Z* phantom from these pair-production electrons/positrons. Phantoms were created with Dragon Skin^TM^ (Smooth-On Inc., Macungie, PA) high performance silicone rubber which is often used for skin prosthetics. Figure 1 shows how this phantom was designed to mimic the upper layers of human skin.

**Figure 2.**
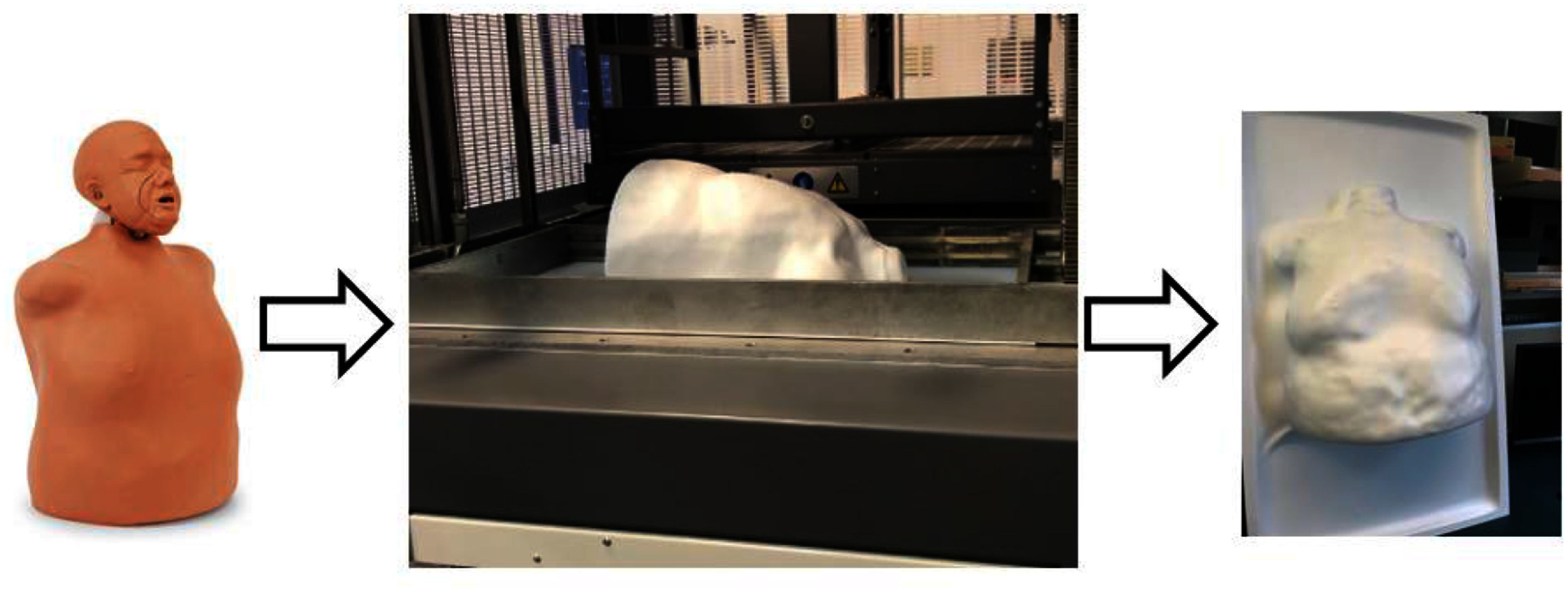
Process of creating the plastic mold of the anthropomorphic CPR phantom, using a thermoformed plastic negative mold.

To form the silicone skin material that was to be draped over the anthropomorphic phantom, an HD-1000c-T vacuum former (Formech, Middleton WI) was used to create a plastic mold. High Impact polystyrene plastic (SKU: 24786, Laird, Sun Prairie WI) was heated and then pressed into the phantom with a vacuum seal formed as indicated in figure 2.

The Dragon Skin^TM^ silicone was mixed with 0.27% by mass of light skin tone pigment with an approximate pantone matching system (PMS) value of 488 C (Silc Pig^TM^, Smooth-On Inc., Macungie, PA) and was then placed in a vacuum chamber (VEVOR, Rancho Cucamonga, CA) to remove the gas bubbles from the mixture. Once degassed, the viscous silicone mixture was poured along the sides of the mold and periodically adjusted to allow the material to cure, forming the various surface features of the phantom. The light skin tone pigment was selected to be the color for the bulk of the material to simulate the color of oxygenated blood vessels in the dermis and subcutaneous layers of skin.

Because melanin is only found in the outer epidermis region of skin, an outer layer of paint was applied to the skin phantoms to imitate this structure. A high-volume low-pressure paint sprayer (C800971, HomeRight, Plymouth MN) was used to apply a thin, homogenous layer of silicone-based paint (Psycho Paint^TM^, Smooth-On Inc., Macungie, PA) that was mixed with paint thinner (NOVOCS^TM^, Smooth-On Inc., Macungie, PA). Various concentrations of black (PMS Black) and dark-skin-tone (PMS 469 C) pigments were used to produce the spectrum of skin-tones. No paint was applied for skin type I, 0.5% concentration by mass of dark skin tone pigment was used for type II, 1.0% for type III, 2.0% for type IV, 5.0% for type V, and 4.6% and 2.9% of dark skin tone and black pigment, respectively, for skin type VI.

**Figure 3.**
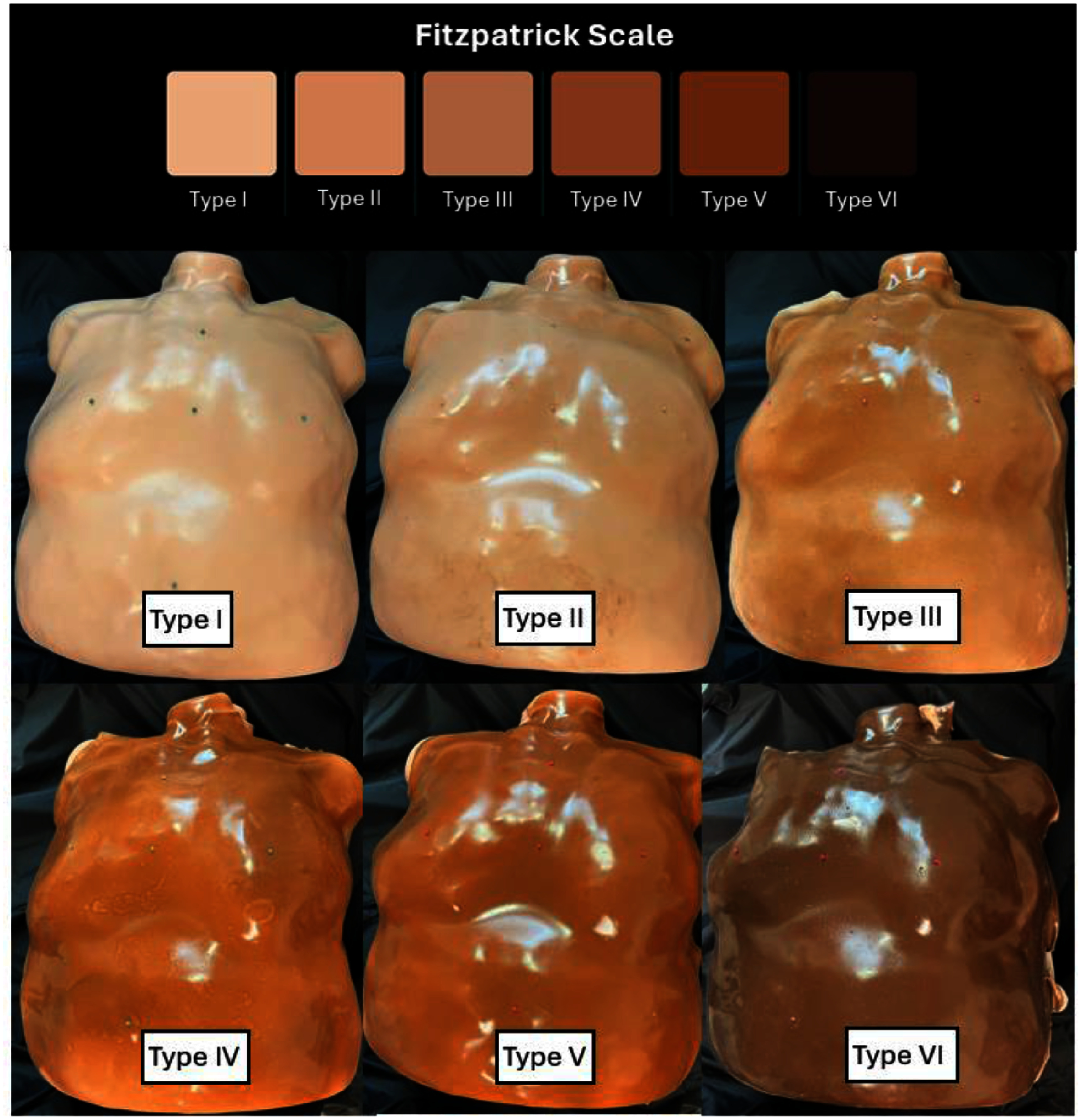
Skin phantoms of various skin tone. Each phantom, nearly identical in shape, was created of silicone mixture in the plastic mold (figure 2). Each was painted separately, with custom silicone paint pigments to match the six skin types listed in the Fitzpatrick scale, types I–VI.

These pigment concentrations were specifically chosen to mimic the Fitzpatrick scale of skin tone, with verification by measurement of the melanin index (MI) of the phantoms, as illustrated in the photos of figure 3.

The Fitzpatrick sun-reactive skin typing scale is typically determined using a questionnaire focused on the patient’s susceptibility to UV light. The skin tone levels range from skin types that easily burn and never tan (type I) to skin that rarely burns and tans easily (type IV) to brown and black skin (types V and VI) (Gupta and Sharma 2019). Although biased towards patients with lighter skin, the Fitzpatrick method has been the primary method for dermatology studies using skin type/color as the independent variable (Gupta and Sharma 2019, Harvey *et al* 2024). The skin typing of the phantoms was based purely on visual perception. To further validate each skin type, the MI and International Commission on Illumination (CIE) CIE-luminosity (CIE-L) values were obtained. MI has been validated in several studies to correlate well with the Fitzpatrick skin scale. Studies have shown that the Fitzpatrick scale and the MI have strong positive association and similarly effective diagnostic capabilities (Wilkes *et al* 2015, Sitek *et al* 2016, Khalid *et al* 2017).

**Figure 4.**
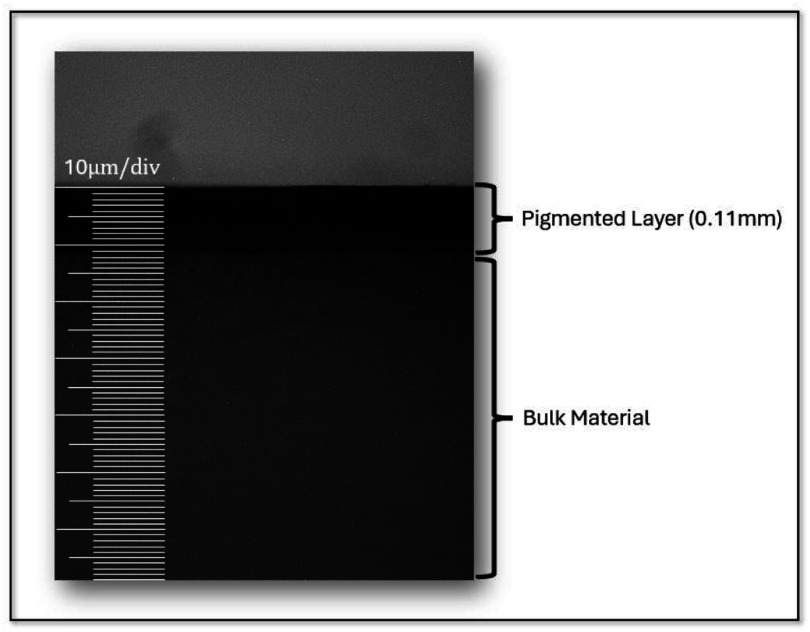
Grayscale, 8x image of a cross section of a sample from one of the tissue phantoms. This sample was found to have a top pigment layer with a thickness of 0.11 mm. On average, the phantoms exhibited top layers with a thickness of 0.12 mm $ \pm $ 0.03 mm.

Small samples were cut from the phantoms and imaged using a SZ stereomicroscope (Olympus, Center Valley, PA) with an OptixCam summit series camera attachment (Microscope LLC, Roanoke, VA). The thickness of the top layer of the phantom was measured using 8x images of the sample and a 0.01 mm per division calibration slide. It was found that the thickness of the top pigment layer was 0.12 mm $ \pm $ 0.03 mm, 33% greater than the epidermal depth of the human abdomen which has a thickness of 0.08 mm (Lintzeri *et al* 2022). This slight increase in thickness is expected to have minimal effect on the experimental relevance to clinical cases. Figure 4 shows an example image of the magnified upper phantom layer.

The MI and the CIE-LAB color space luminosity value (CIE-L), were determined using a DSM-4 colorimeter (Cortex Technology, Aalborg Denmark). 20 measurements were taken for each phantom while pressing the colorimeter against the phantom surface with the results averaged. The MI is calculated as,

\begin{align*}\begin{array}{*{20}{c}} {{\text{MI}} = 100 \times {{\log }_{10}}\frac{1}{{{R_{\text{r}}}}}} \end{array}\end{align*}

where ${R_{\text{r}}}$ is the reflectance measured at a wavelength of 680 nm. It should be noted that the MI values recorded do not perfectly correspond with those seen in literature for the Fitzpatrick skin types (Wilkes *et al* 2015), however, the skin type terminology was adopted for ease of presentation. The CIE-L (luminosity), on the other hand, is a relative lightness scale ranging from 0 (black) to 100 (white).

Additionally, measurements of the phantom’s diffuse reflectance were made using a U–VIS Lambda 19 spectrophotometer. Small cylindrical versions of the phantoms were produced such that they would fit inside the spectrophotometer for the measurement. A reference measurement was made using a white standard and was then used to normalize the phantom measurements. The diffuse reflectance is reflectance off a surface where the light is scattered in multiple directions, which is expected when light travels through the phantom material (Clark 2006). The U–VIS Lambda 19 is designed to collect this scattered light using an integrating sphere.

### 2.2. Treatment planning

A secondary hypothesis in this work was that the treatment plan modulation would affect the measured light, with the most modulated plans having the least light output. Additionally, more modulated plans require more monitor units (MUs) which may result in higher integral dose, producing signal outside of the designated beam (Craft *et al* 2007). A total of 18 left breast treatment plans were created—three for each skin tone phantom—to compare how well the Cherenkov emission can be localized for each external beam treatment type across all skin tones.

Left breast patients are often treated utilizing a deep inspiration breath hold technique which spares the heart by pulling it away from the chest wall [16, 17]. Therefore, an immobile phantom can emulate a clinical scenario effectively. A CT simulation was performed for each phantom in collaboration with the UW Health Department of Human Oncology to ensure phantom position reproducibility. Push pins used as fiducial markers were placed on the phantom along the external positioning lasers to mark the tissue phantom’s location relative to isocenter.

CT scans were imported into RayStation (RaySearch Laboratories, Version 11B) and three plans were produced for each of the six variable skin tone phantoms: (1) static tangent beams, (2) field-in-field, and (3) volumetric modulated arc therapy (VMAT). The tangent field plan was chosen to demonstrate a simple plan with open fields and minimal blocking. The field-in-field plan acted as a slightly more complex example where truncated fields are utilized in conjunction with open fields to aid in lowering high dose regions. The VMAT plan was used to test the efficacy of Cherenkov imaging with complex planning where small, dynamic leaves modulate much of the dose in an arc arrangement. All 18 plans were loaded into the C-dose research software (DoseOptics LLC, Lebanon NH) which estimated an image of the expected surface dose in the top several millimeters of skin. The software was then used to compare with the actual imaged Cherenkov.

### 2.3. Treatment delivery imaging

Treatment delivery was carried out on a TrueBeam linear accelerator (Varian Inc, Palo Alto CA), and real time imaging of the Cherenkov emission was achieved by a BeamSite® Research camera (DoseOptics LLC, Lebanon NH) using the BeamSite Research Software version 5.01.04. To ensure that the imaged Cherenkov light aligned with the virtual 3D space of the C-Dose research surface dose visualization, an extrinsic camera calibration was performed to determine the camera position relative to the linac isocenter. All plan/phantom combinations were imaged three times with the camera setup at a different angle each time. The camera-to-isocenter distance was roughly 1.5 m for each acquisition.

An anterior–posterior (AP) field was used to irradiate each phantom with the BeamSite camera mounted above to demonstrate the linearity between the MI and luminosity versus the detected light intensity. A region of interest (ROI) was drawn on each phantom’s breast and the average pixel value in each phantom ROI was calculated along with the standard deviation normalized to the corresponding ROI on the type I phantom. The variance in the various ROI mean values was recorded as the error bar. To make comparisons between the planned and imaged Cherenkov signal, the phantoms had to be accurately positioned on the linac treatment couch. For this set up, each phantom was positioned using push pins placed during the CT simulation and the linac kilovoltage cone beam CT was used to image the phantom and align it with the plan. Video rate Cherenkov images were then recorded.

The frame rate is dependent on dose rate due to the camera acquisition being triggered by the pulses of the linac. For a static, 600 MUs per minute beam, the camera frame rate is expected to be approximately 36 fps, however, the framerate for VMAT plans is harder to calculate due to the inconsistency in the pulse rate throughout the treatment. Individual frames were added together and the resulting cumulative images were compared to the surface dose estimation calculated from the treatment plan dose and structure files. The dice-similarity-coefficient (DSC) acted as the dependent variable and was determined for every combination of phantom skin-tone and treatment plan. DSC is calculated as follows,

\begin{align*}\begin{array}{*{20}{c}} {{\text{DSC}} = 2\frac{{\left| {A{{\mathop \cap \nolimits}}B} \right|}}{{\left| A \right| + \left| B \right|}}} \end{array}\end{align*}

where *A* is the region of the image expected to contain Cherenkov light based on the treatment plan and *B* is the actual measured Cherenkov region.

The DSC value is dependent on the intensity threshold values set for the Cherenkov image as well as the treatment plan surface dose estimate where pixel values below the threshold are ignored. A plan threshold of 30% of the max dose hotspot was fixed for all comparisons and the image threshold was iterated from 15% to 45% using the analytics feature within the BeamSite software. The largest DSC value obtained using this parameter search method was tallied. This analysis was carried out for data acquired with the room ambient lights off and was repeated 3 times for each phantom and plan to avoid any statistical outliers from human error. For each repeated treatment, the phantom was removed from the couch and re-aligned, ensuring the setup method was robust. The Tukey honestly significant difference test was conducted to determine the statistical significance of the degradation in plan quality observed in the VMAT treatments for the darkest skin tone.

## 3. Results

### 3.1. Phantom skin color verification

**Figure 5.**
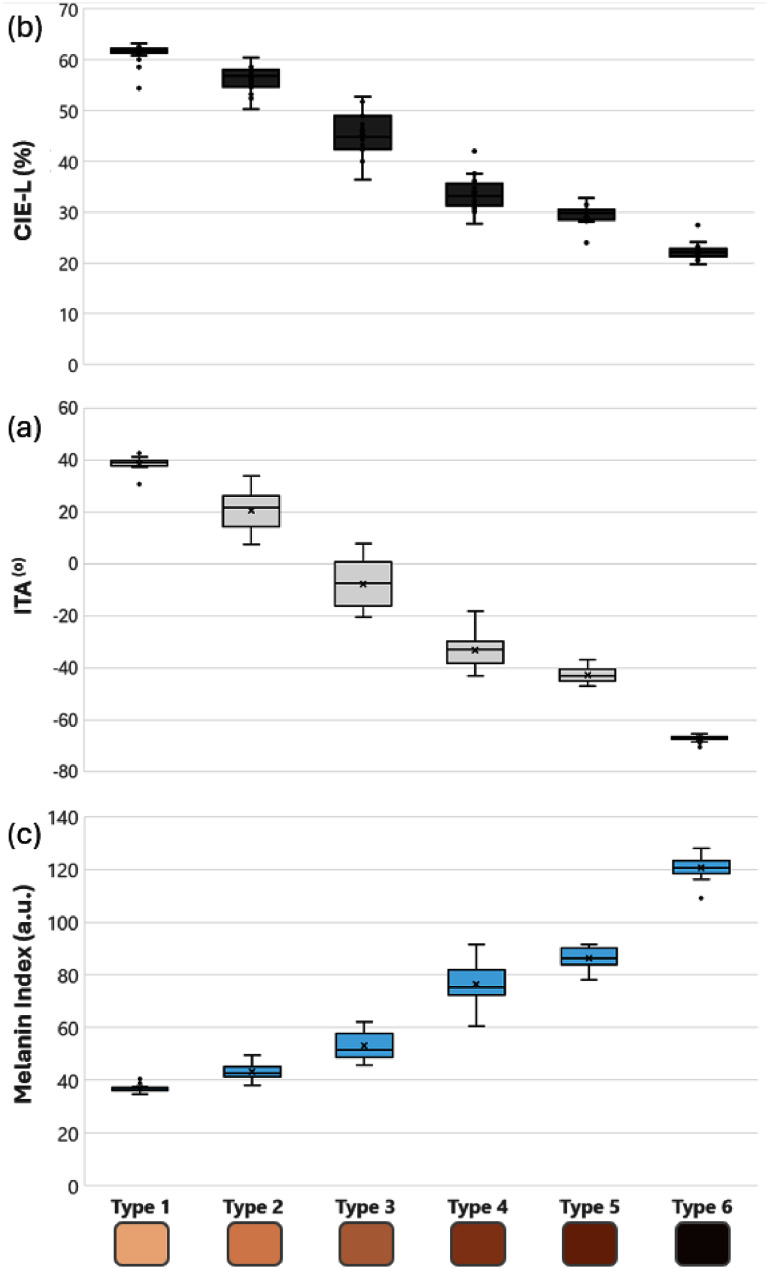
(a) CIE-luminosity (CIE-L) measurements for all 6 phantoms using the colorimeter. Values are listed in percentage with 0% corresponding to pure black and 100% for pure white. (b) ITA measurements for each skin type where ITA is calculated using the L* and b* values from CEI color space (c) melanin index measurements for each phantom across the Fitzpatrick scale of skin types.

MI, CIE-L, and the individual typology angle (ITA) were measured in all phantoms in approximately 20 locations on each phantom, around the abdomen and breast regions. Figure 5 summarizes the mean pigmentation level while the error bars (standard deviation) represent the homogeneity of the pigmentation. The average values were used as the independent variables for the study. No consensus range of MI levels exist for determining the Fitzpatrick skin type (Wilkes *et al* 2015), likely because there is no standard method for where on the body to measure MI. Regardless, the increase in MI and decrease of CIE-L and ITA across the reproduced phantom skin types showed a reasonable range of these metrics for each skin type.

**Figure 6.**
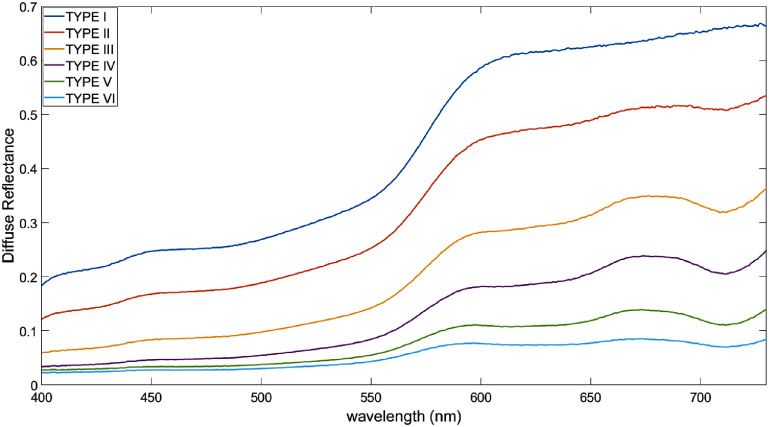
Diffuse reflectance measurements of skin type phantoms made with a white standard reference. Data was collected using a U–VIS Lambda 19 spectrophotometer. For darker skin types, the reflectance decreased as expected but kept a similar spectra trend including skin type VI which had a small concentration of black pigment added to the top layer.

The diffuse reflectance spectral measurements made with the U–VIS Lambda 19 spectrophotometer are displayed in figure 6.

The resulting spectra for skin types II–VI demonstrate a stronger reflectance in the large wavelength range as expected for pigments designed to replicate melanin. Measurements of human skin made by the National Institute of Standards and Technology demonstrate the same trend where greater diffuse reflectance is observed for larger wavelengths with the signal dropping below a wavelength of 600 nm (Cooksey *et al* 2017, Mignon *et al* 2018). Other studies in the literature show similar results with the greatest discrepancies occurring in the short wavelength range (<600 nm) (Zonios *et al* 2001, Borisova *et al* 2018). These differences between data sets can be ignored, however, because the quantum efficiency of the Cherenkov camera is heavily weighted towards the red and infrared region of the spectrum, making the disagreement between the measurements from this study and literature below 600 nm inconsequential. The shape of the spectra is consistent as the concentration of pigment in the paint is increased.

### 3.2. Cherenkov imaging versus MI and CIE-L

**Figure 7.**
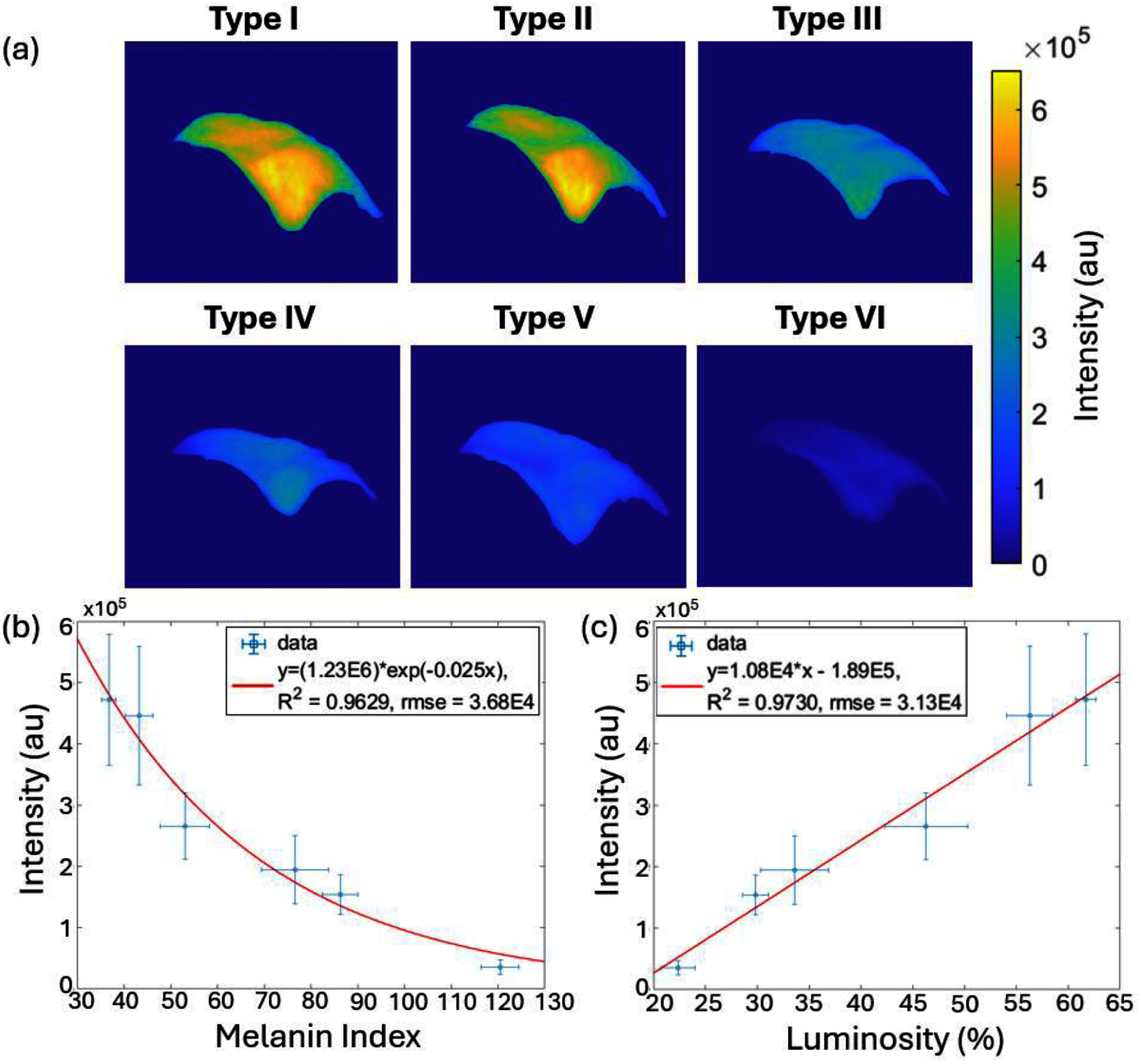
Linearity of Cherenkov signal with CIE-L percentage and melanin index. In (a) images from each of the phantoms are shown with varying skin melanin index. Average intensity values from the breast ROIs are plotted vs melanin index (b) and CIE-luminosity (c). The fit to the data in (b) was modelled by exponential decay because of the expected diffusion from the optical attenuation coefficient which is linear with MI. The relationship between intensity and luminosity in (c) was assumed to be linear.

Images of a Cherenkov ROI of the uniform phantom irradiation can be seen figure 7(a). Analysis of this AP field data demonstrated that the relationship between CIE-L and the measured Cherenkov intensity was linear in the range of color values used with an *r*^2^ value of 0.97, while the relationship between the MI values and intensity followed an inverse exponential trend with an *r*^2^ value of 0.96. The luminosity is related to the amount of light scattering in the phantom and escaping the surface to be detected by the colorimeter, while the Cherenkov camera measured the amount of diffuse Cherenkov light escaping the phantom. We expect a linear relationship between these values within the range measured. Additionally, the effective attenuation coefficient of the phantom can be assumed to be linearly dependent on MI. It was shown by Decker *et al* that according to diffusion theory, the radiant emission of Cherenkov should have a nearly inverse exponential dependence on the effective attenuation coefficient(2021). The plots of Cherenkov intensity as a function of MI and CIE-L are displayed in figures 7(b) and (c) respectively. The match between the data and expected trend were felt to be sufficient to allow for proceeding with all treatment plans and imaging, given that the phantoms represented a range of tissue MI levels and that the emitted Cherenkov appeared to show linearity with the measured luminosity.

### 3.3. Treatment imaging

**Figure 8.**
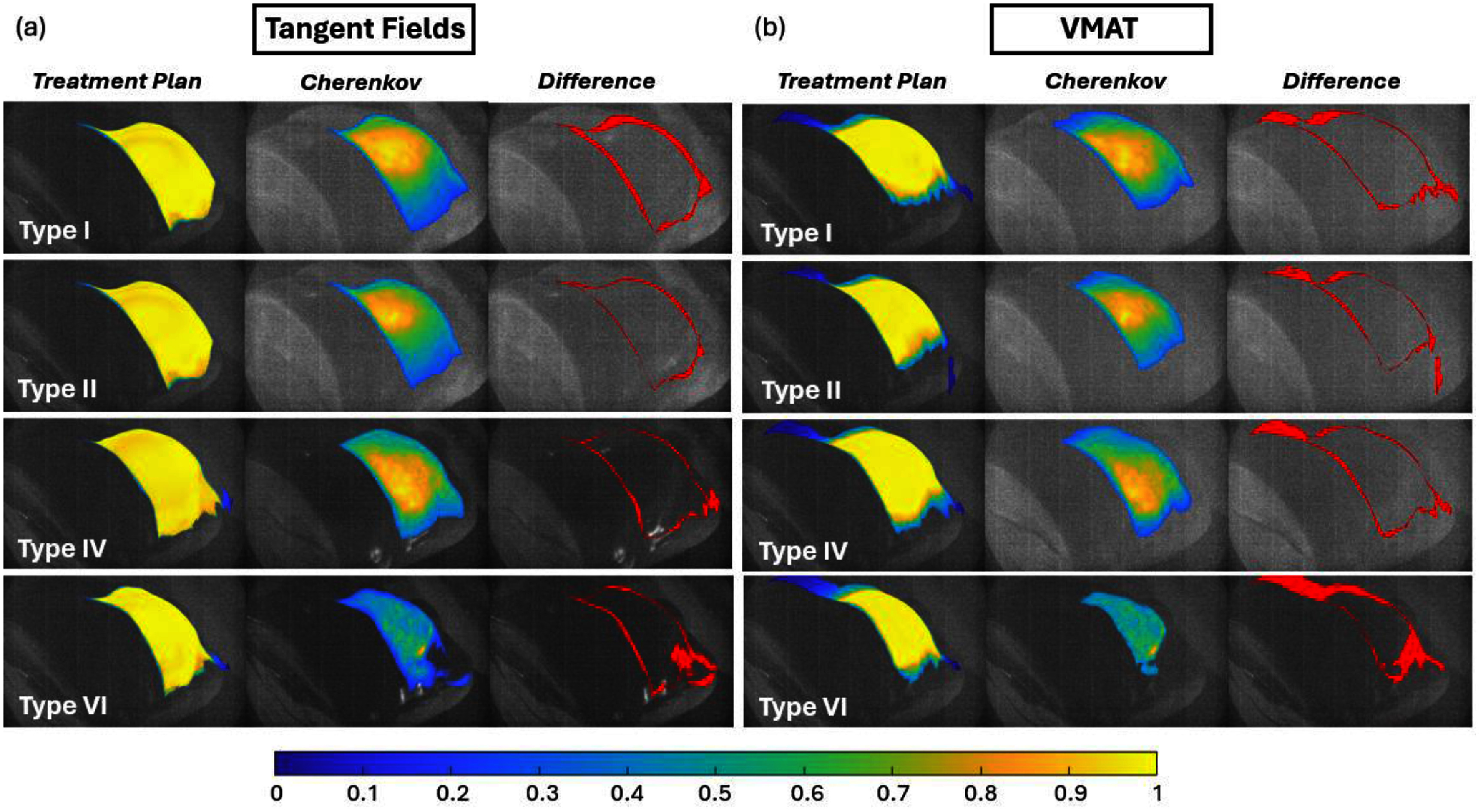
Surface dose, Cherenkov images, and binary difference maps calculated from the plan and Cherenkov signal across 4 of the analyzed skin types on a thoracic phantom. (a) Shows the tangent field plans while (b) shows the VMAT images. Cherenkov signal (middle column) is shown beside the estimated planned surface dose (left column). Types III and V as well as FIF treatments were omitted for brevity.

Figure 8 demonstrates that across all skin types, good agreement exists between the planned and imaged surface dose except for the darkest skin tone with skin types I–V demonstrating DSC values of ∼95%. When considering static fields for the breast tangents, the main factor limiting the emission of Cherenkov light is absorption at higher melanin concentration. Although more melanin can affect the dosimetry capabilities, it does not affect the ability to localize beam shape until reaching a MI of 120 and a CIE-L of 22.4% (Skin type VI). Even when considering type VI, the mean DSC was ∼88%. The field-in-field plans showed very similar results. These plans are nearly identical to the tangent field plans except for the presence of smaller beamlets applied near the end of the treatment delivery to minimize dose hot spots. Although this introduces potential for Cherenkov inaccuracies, all phantom images showed excellent beam shape accuracy. Even skin type VI (MI = 120), showed good agreement with an average maximum DSC of 88%.

VMAT plans show a larger drop in image intensity compared to the less modulated plans for the darkest skin type when considering the DSC values. Like the previous plans, skin types I–V perform well according to the DSCs, but a substantial reduction (>10%) in accuracy was observed with type VI as compared to the other plans. It is evident in figure 8 that for type VI skin renderings, the Cherenkov light begins to drop to an intensity close to the out of field signal which is made of image noise and Cherenkov produced from leakage radiation. This results in a drop in the plan to Cherenkov geometric accuracy. The max dice score for this scenario was recorded between 75% and 80%.

Sample images of the tangent field plan Cherenkov images are depicted in figure 8 along with their corresponding estimated plan dose. The difference column shows the binary difference map between the planned surface dose and the Cherenkov signal. The threshold values used to produce the binary difference map were the same as those used for calculating the DSC (defined in section 2.3). The imaged and planned data varies at high melanin concentration (type VI). However, the difference maps (figure 8, right column) show that as the skin tone gets darker for both the tangent field plans and the VMAT plans, the deviation from plan is not overtly worse for the VMAT compared to the tangent plan until reaching the darkest skin tones.

**Figure 9.**
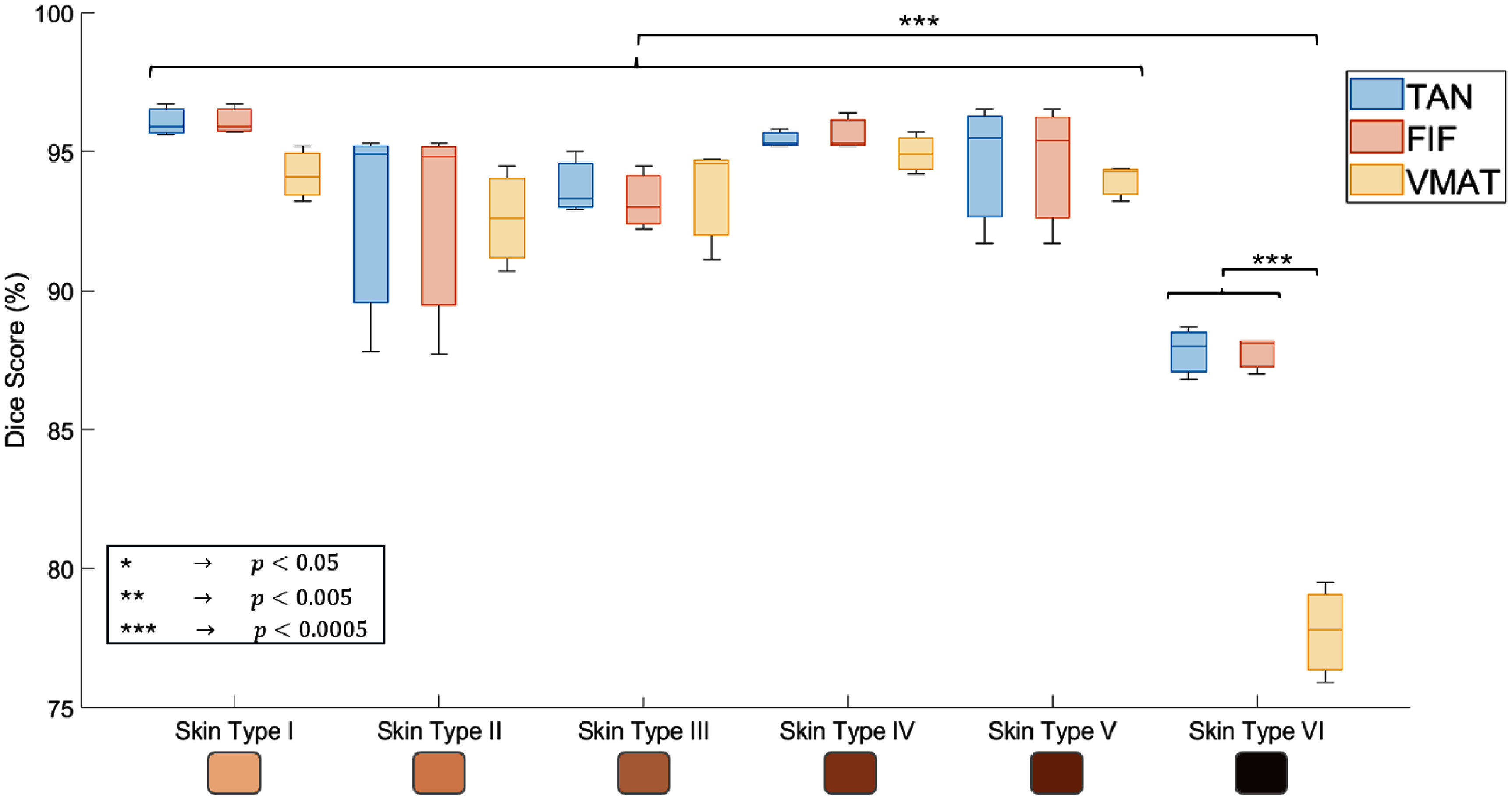
Dice scores for all 18 treatment plans compared to the imaged Cherenkov signal demonstrating stable performance across skin tones except for type VI. Note how the *y*-axis goes from 75% to 100%.

The complete set of DSCs are summarized in figure 9. Across all phantoms and treatments when considering several images of the same phantom with repeated setup, the average standard deviation of the DSC was 1.5% pointing to good setup reproducibility. As mentioned above there is excellent agreement between the planned and imaged dose with fluctuations likely arising from slight phantom irregularities and positioning errors. However, each set of plans demonstrates a drop in DSC at high MI (low luminosity), with the VMAT plans showing the greatest DSC decrease. It was determined that there was a statistically significant difference between the dice scores for skin type VI relative to all others (*p*-value greater less than 0.005) except for when comparing the tangent and FIF plans for skin type II to the same plans for skin type VI. No statistical significance was found when comparing all combinations of skin types and plans between types I and V. The VMAT plan for skin type VI was found to have a *p*-value less than 0.0005 when compared against all skin type and plan combinations. Specifically, comparing the skin type VI VMAT plan to the FIF and tangent plans for the same type resulted in *p*-values of 2.5 × 10^−5^ and 2.8 × 10^−5^, respectively.

## 4. Discussion

This work sought to develop and validate a series of variable skin phantoms and perform a sensitivity test of Cherenkov acquisitions by radiotherapy technique. While several clinics are now using Cherenkov imaging *in vivo*, there are several factors that make phantom imaging ideal for these types of studies. Phantoms can be made to be very similar in shape and bulk material to avoid major deviations in imaged subjects across the skin type range. This can allow for a more focused investigation of specific variables related to the subject, in this case, the observed skin tone when compared to the Fitzpatrick skin scale. Furthermore, because there is no additional goal of treating cancer, the phantoms can be irradiated with nearly identical plans allowing for a more controlled study. The lack of radiation limits for phantom studies allows for re-irradiation, obtaining multiple images and subsequent statistical analysis. Dosimetrically, silicone acts as a great surrogate for human tissue as the densities closely match. Additionally, the effective atomic number of silicone is close to that of tissue although slightly larger.

The use of a tissue phantom that accurately simulates the way that light travels through tissue as a diffusion process is critical to interpreting Cherenkov imaging as the light attenuation of melanin occurs in the most superficial layers of the epidermis. The phantoms produced here were successful in this regard, as measured values of CIE-L decreased with the skin types measured while MI decreased. The Cherenkov intensity varied linearly with the measured CIE-L scale values while decreasing exponentially with the MI as would be expected for human tissue of increasing MI. Importantly, radiotherapy tissue phantoms that have less translucency to them and/or have the absorber embedded throughout the tissue volume would likely not mimic the expected emissivity with MI suggesting that these optical phantoms are suitable surrogates for patients.

The phantoms were further validated using the diffuse reflectance measurements shown in figure 6. While not perfect at mimicking the spectra of melanin, the pigments used in this work were designed to closely match the pigment of skin as they are commonly used for prosthetic makeup. Our measurements validate that within the spectral sensitivity range of the camera, the diffuse reflectance signal is largely uniform as would be expected for skin with melanin.

Cherenkov surface imaging is a qualitative tool, not used for correlating exact dose delivery to the measured intensity, but instead used to provide spatial information about where dose is deposited. This allows physicians to determine if the appropriate regions of the patient are being treated. Loss in accuracy of the treatment field shape might be misinterpreted for a loss of full dose coverage in the treatment areas. Clinical implications of the findings of this study demonstrated an apparent signal loss that might occur in the darkest skin tone subjects. Published work by Zhang *et al* demonstrated a decrease in signal across typical human skin tones by nearly a factor of 10 consistent with this work (2017). This loss of signal is especially apparent at the edge of the treatment field due to this being the lowest intensity area, and this can be compounded by an overall lower Cherenkov intensity per frame captured during VMAT treatments.

The light signal detected on the patient surface outside of the direct beam is due to a combination of head leakage and patient scatter producing Cherenkov in the phantom, ambient light in the linac bunker, as well as dark current camera noise. All these sources result in a greater detected ambient signal relative to the Cherenkov signal for VMAT compared to the tangent and field-in-field plans. This is because VMAT requires a larger number of MUs to deliver the same prescription dose which ultimately results in greater out of field dose and a greater number of accumulated frames, ultimately leading to the collection of more ambient light and dark current counts although not included in this work, Monte Carlo simulations of emitted Cherenkov light calculated using the patient CT and plan parameters could help predict these out of field light sources. For skin-types I–V, this out of field signal is negligible relative to the Cherenkov light detected where the beam interacts with the patient. This is not the case for the darkest phantom. The nature of VMAT to produce less Cherenkov signal from small, modulated beams, mixed with the increase of background signal on and around the patient reduces the contrast between the beam and its surroundings.

Studies assessing the MI of the inner portion of the upper arm of patients across various global populations have demonstrated that the darkest human skin exhibits MI values around 125 (Crawford *et al* 2017, Dadzie *et al* 2022). Therefore, the findings presented in this study are applicable as the highest MI of the phantoms was measured to be 120. Thus, finding techniques to increase the amount of Cherenkov light captured for all patients must be developed to ensure equitable use of Cherenkov imaging. Despite this need, most patients more closely match skin types I–V, suggesting the current clinical implementation of Cherenkov imaging provides meaningful results for all studied treatment types in most cases.

As this data demonstrates, Cherenkov imaging provides meaningful dose localization data for all skin types except for the type VI demonstrating DSCs < 90%. Although a useful metric, threshold DSC values for Cherenkov imaging have not yet been defined. Adjacent applications of the DSC metric, such as physician contoured organs for treatment planning, can supply a benchmark. According to Woerner *et al*, the mean physician to physician dice score for all organs was 90% with some organs showing 84% DSC (2017). Therefore, it is reasonable to assume that DSC around 90% should be the goal for Cherenkov imaging, however, in the case of the darkest skin tones, the DSC is lower due to a loss of signal near the field edges despite the potential that the radiation is properly delivered. A DSC value of 80% is still valuable for general dose localization, but lacks the complete information of the radiation field’s extent.

While all treatment plans for skin type VI resulted in DSC < 90%, VMAT demonstrates the greatest discrepancy between planned dose and the imaged Cherenkov light and should be the focus for future work. It should be noted that the increase in effective atomic number when comparing silicone phantoms to skin tissue slightly increases the number of pair-production events in the medium which may result in a slight increase in Cherenkov light at the surface of the material, although the magnitude of this is not thought to be highly significant here due to the energy range of radiotherapy.

When considering the imaging of clinical treatments on patients with high melanin content, techniques for improved light collection should be investigated to ensure maximum match to the surface dose map. These techniques could include the capture of more light with a lower f-stop lens or altering the signal to noise ratio of the camera to increase the signal captured per unit frame. It may be possible to design an adaptive imaging protocol where the frame rate of the camera or the signal to noise is specifically increased when a patient with darker skin is seen in the image. Alternatively, reduction in room lighting can also help in VMAT treatments because of the increased number of frames being added together for a cumulative image, which inherently adds in more background light leakage into the signal. The data in this study was collected with the room lights off, verifying that clinically useful images can be obtained for darker skin if these conditions are replicated in the clinic. While further *in vivo* validation is needed to confirm these findings, results obtained in this work can help guide clinical Cherenkov interpretation with existing clinical systems that are in use.

## 5. Conclusions

Cherenkov imaging is a very useful tool for surface guided radiation therapy, but up to now the ability to spatially localize treatment on patients with very dark skin tones had not been sufficiently studied. The results presented in this work demonstrate an expected reduction in signal across skin tones. However, the findings from this work highlight the clinical capabilities of this technology even when treating the darkest skin tones as Cherenkov light remains visible, outlining a significant portion of the treatment field. However, while the DSC remains greater than 90% for skin types I–V, skin type VI experiences a ∼10% drop for the FIF and tangent plans and a greater than 10% drop for the VMAT plans. Therefore, although the spatial correlation is reasonable, careful interpretation of data collected from patients with MIs nearing 120 should be made, especially when considering highly modulated techniques such as VMAT.

The development of tissue phantoms of various MI provides a useful method for testing imaging techniques designed to counter the loss of light observed for patients with high melanin concentration. More systematic studies should be conducted with these tools to analyze all possible factors that could diminish treatment visualization quality for patients of all demographics. Furthermore, future studies will use Monte Carlo calculations of Cherenkov as a secondary predictive tool and should be followed by a clinical study monitoring patients with varying skin tones. Such studies are necessary to properly assess the true human prevalence of this issue in radiation therapy and test methods for improved imaging.

## Data Availability

The data that support the findings of this study are openly available at the following URL/DOI: https://data.mendeley.com/datasets/36jpf59c9p/1.

## Code, Data, and Materials Availability

• Cherenkov data underlying the results presented in this paper exceed storage limits of most repositories and are therefore available upon request to the author jehallett@wisc.edu.
